# Supplementary material for: Mitochondrial-nuclear cross-talk in the human brain is modulated by cell type and perturbed in neurodegenerative disease
Source: Commun Biol. 2021 Nov 4;4:1262. doi: 10.1038/s42003-021-02792-w (PMC8569145; doi:10.1038/s42003-021-02792-w)
Supplement: Supplementary file 2 — Supplementary Information [file 42003_2021_2792_MOESM2_ESM.pdf]

Supplementary figure 1. Euclidean clustering of 12 GTEx CNS regions based on the Spearman rho of their mitochondrial-nuclear correlation distributions. Spearman correlation (text on squares) represents correlation between 15001*13 mitochondrial-nuclear pair distributions of two CNS regions.

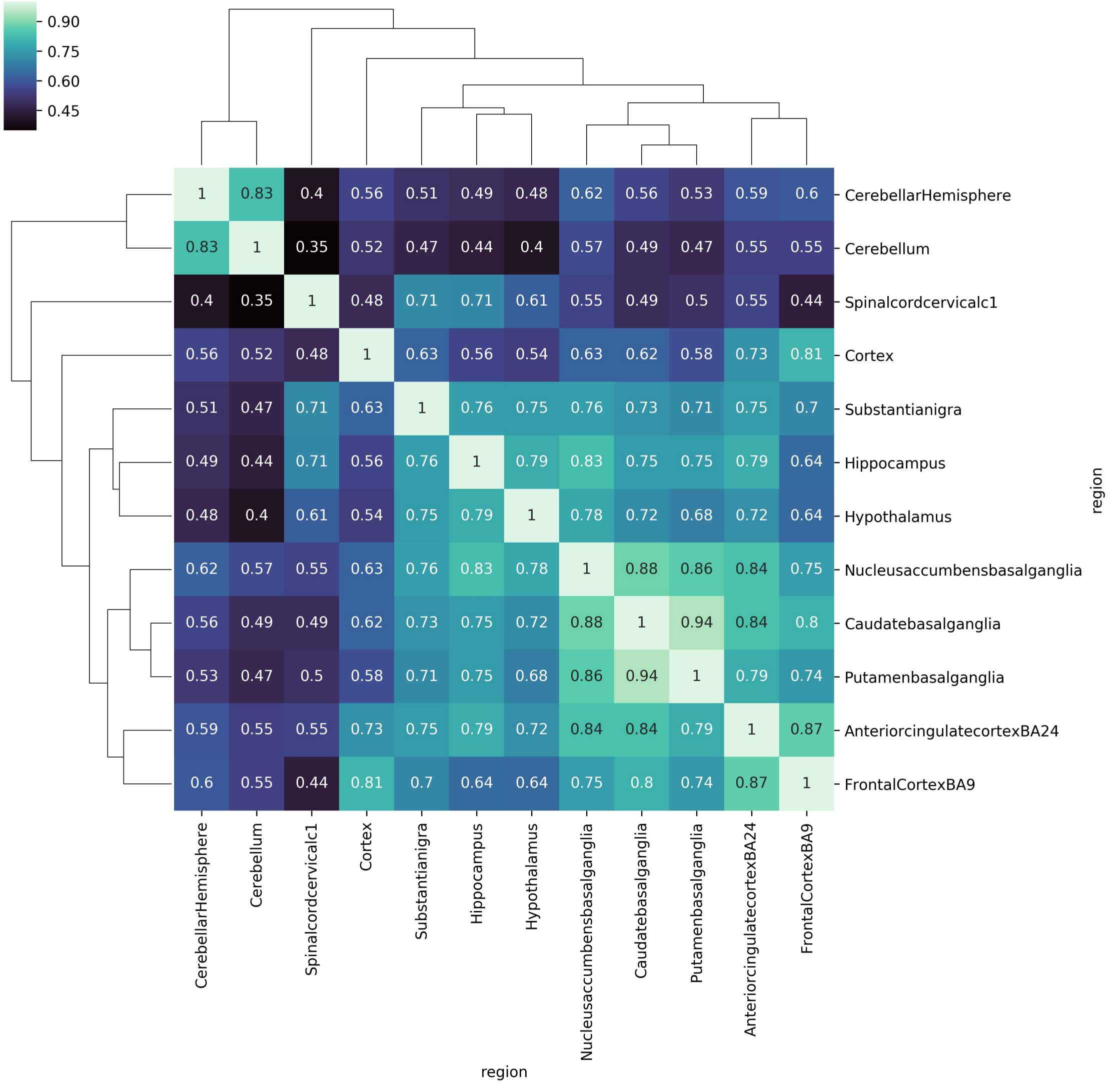

Supplementary figure 2. Figure to illustrate the effect of cell-type correction on mitochondrial-nuclear correlation distributions. **A.** Heatmap to show correlations between PEER factors (generated from TPM values) and estimated cell-type proportions for five GTEx CNS regions selected to be best represented by these estimated cell-type proportions. Annotations correspond to Spearman's rho values. **B.** A barplot to summarise part A, the median PEER cell-type correlation value for each PEER factor is shown for the same five CNS regions. **C.** Distribution of gene-pair (195000 mitochondrial-nuclear pairs) variances across the five GTEx CNS regions for both correction strategies (standard and standard-cell-type). A Wilcoxon signed rank test was carried out to test the null hypothesis that the median of the celltype-standard corrected correlation data was not less than that of the standard corrected data (P < 2.2e-16). Boxplots display the median, upper quartile (Q3) and lower quartile (Q1), with whiskers extending to Q3 + 1.5*IQR and Q1– 1.5*IQR. Diamond points represent outliers above Q3 + 1.5*IQR or below Q1– 1.5*IQR. **D.** Density plot for the five selected CNS tissues, facetted by correction, showing the distribution of nuclear-mitochondrial correlation values under each correction strategy.

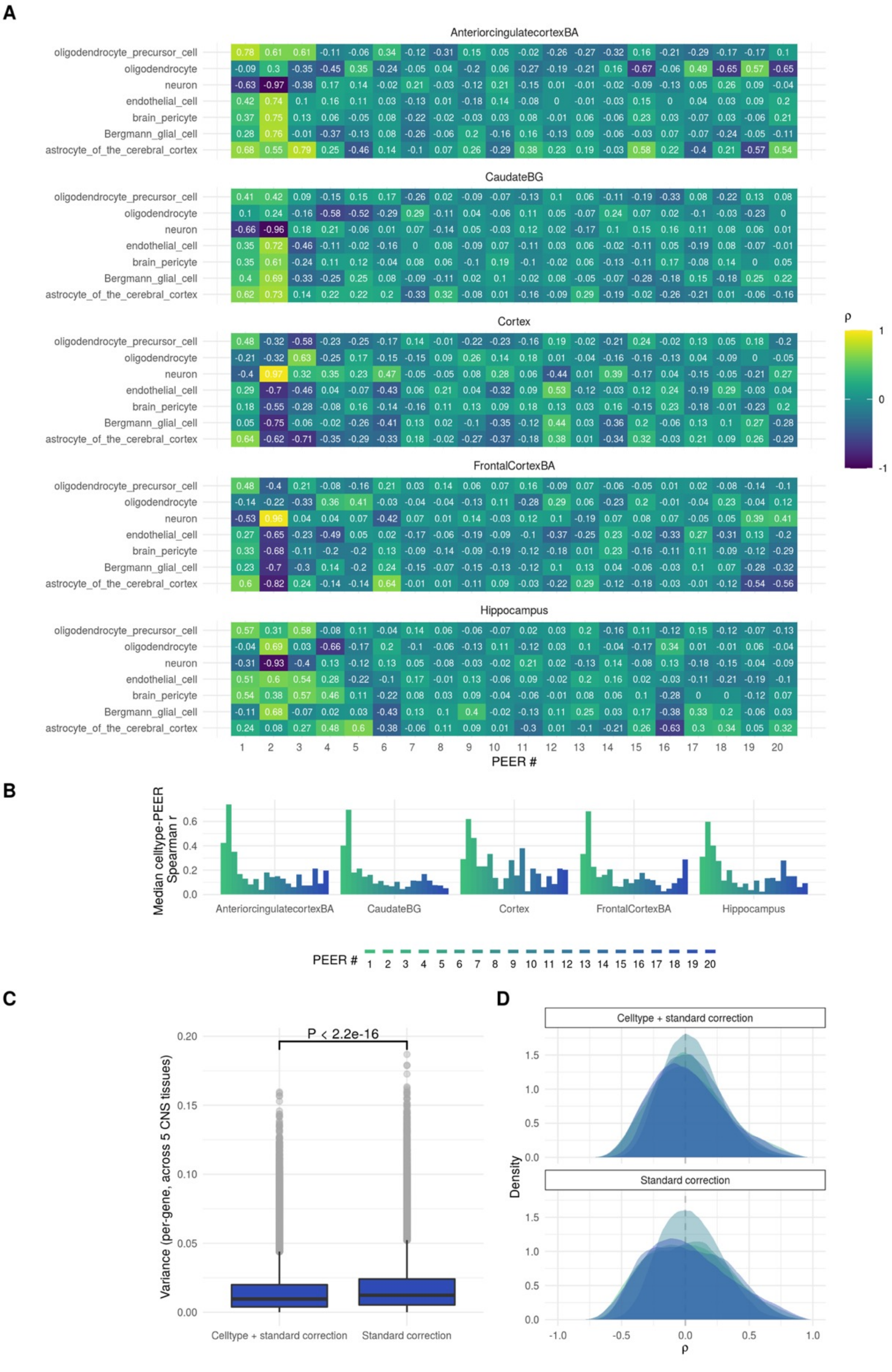

Supplementary figure 3. Plots to show regional distributions of mtDNA gene residuals and intra-genome correlations. **A.** Per-mtDNA gene TPM residuals, coloured by region. **B.** Distribution of nuclear-mitochondrial correlation variances across mtDNA genes for each nDNA gene, grouped by region. Boxplots display the median, upper quartile (Q3) and lower quartile (Q1), with whiskers extending to Q3 + 1.5*IQR and Q1– 1.5*IQR. Diamond points represent outliers above Q3 + 1.5*IQR or below Q1– 1.5*IQR.

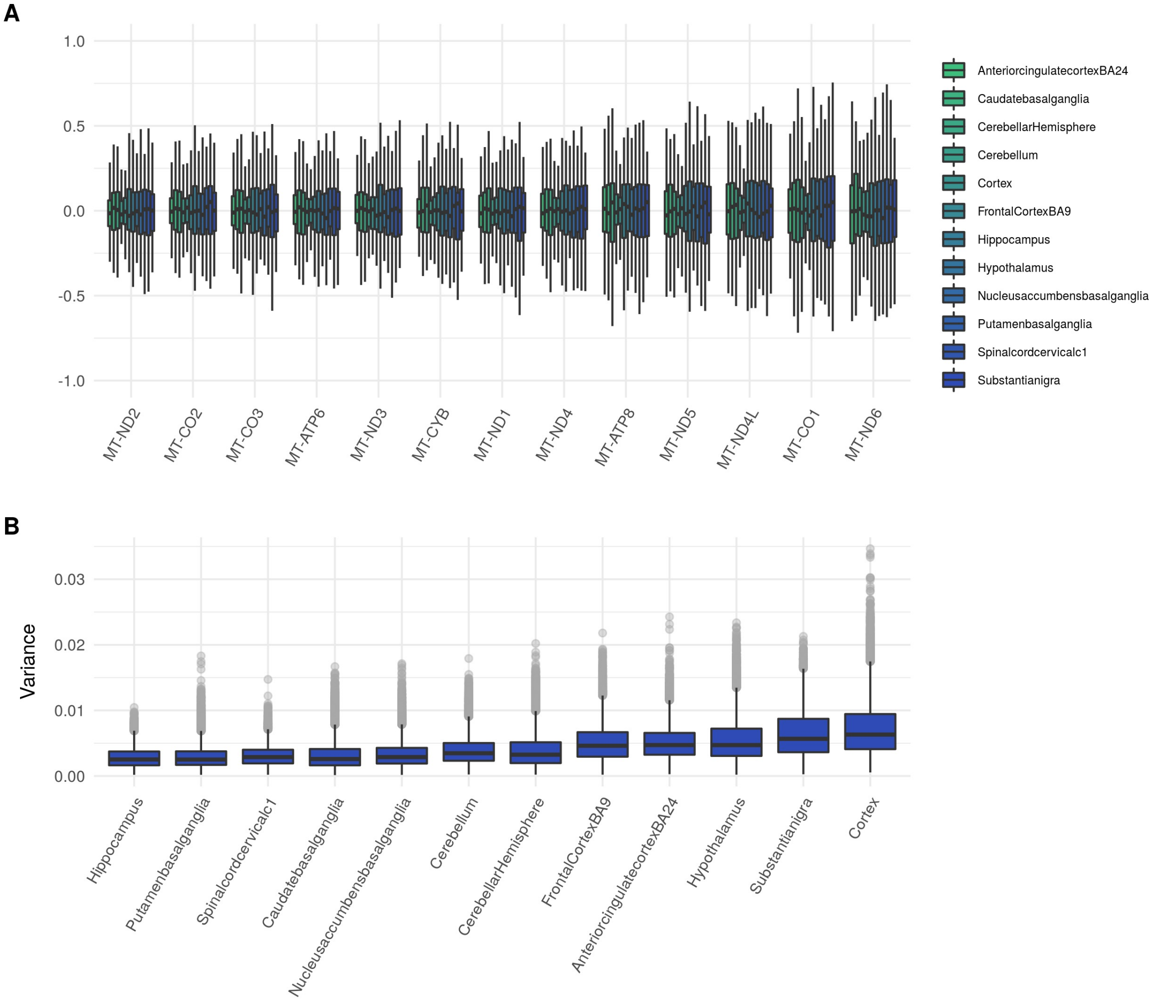

Supplementary figure 4. Analysis to determine whether ND-related gene sets have non-random associations with the mitochondrial genome. **A.** UpSet plot to show the overlap between the four gene sets included in the ‘primary’ disease gene analysis (see methods for gene set details). **B.** UpSet plot to show the overlap between gene sets included in the ‘secondary’ disease gene analysis (see methods for gene set details). **C.** Heatmap to show P-values associated with the median of 11 ND-related gene sets being more extreme than that of 10,000 random gene sets in 12 GTEx CNS regions (* 0.05/12 < P < 0.05; ** 0.05/12*7 < P < 0.05/7; *** P < 0.05/12*7).

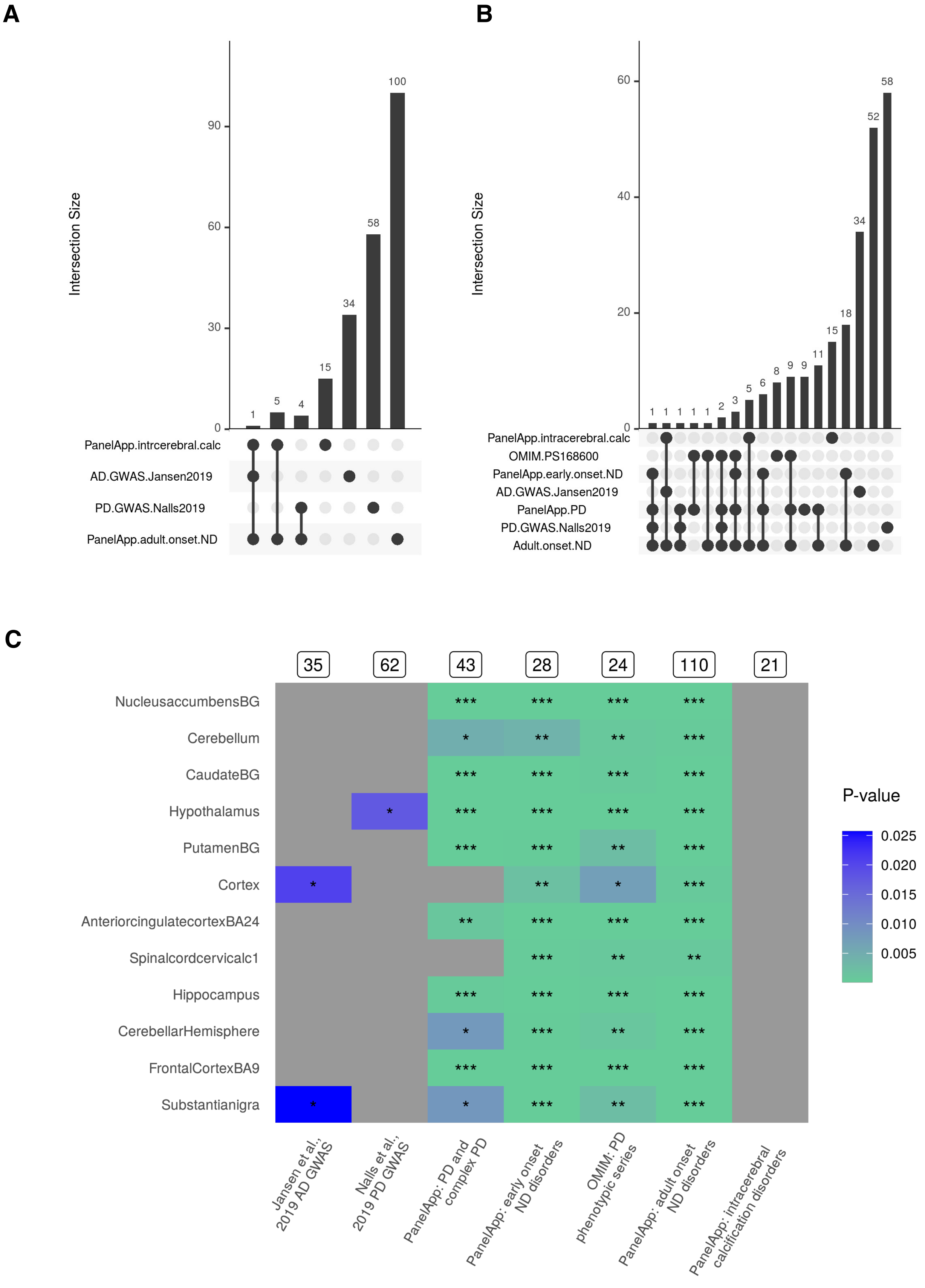

Supplementary figure 5 A. Heatmap output from the MitoNuclearCOEXPlorer tool to show the correlation strength and significance of PSAP to 13 mitochondrial genes. **B.** Plot to highlight the PSAP gene. Ctrl_r represents the range of mitochondrial-nuclear correlations (13 data points) in ROSMAP control data. Case_r represents the the spread of the same13 gene correlations but in ROSMAP case data (AD affected).

A

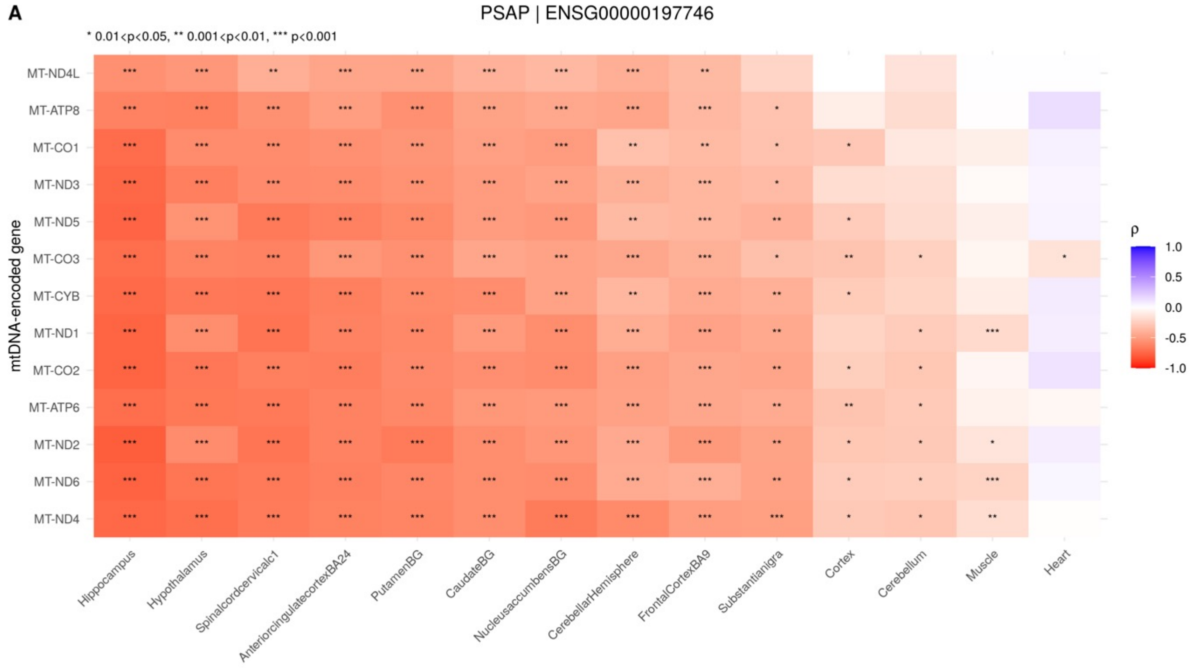

B

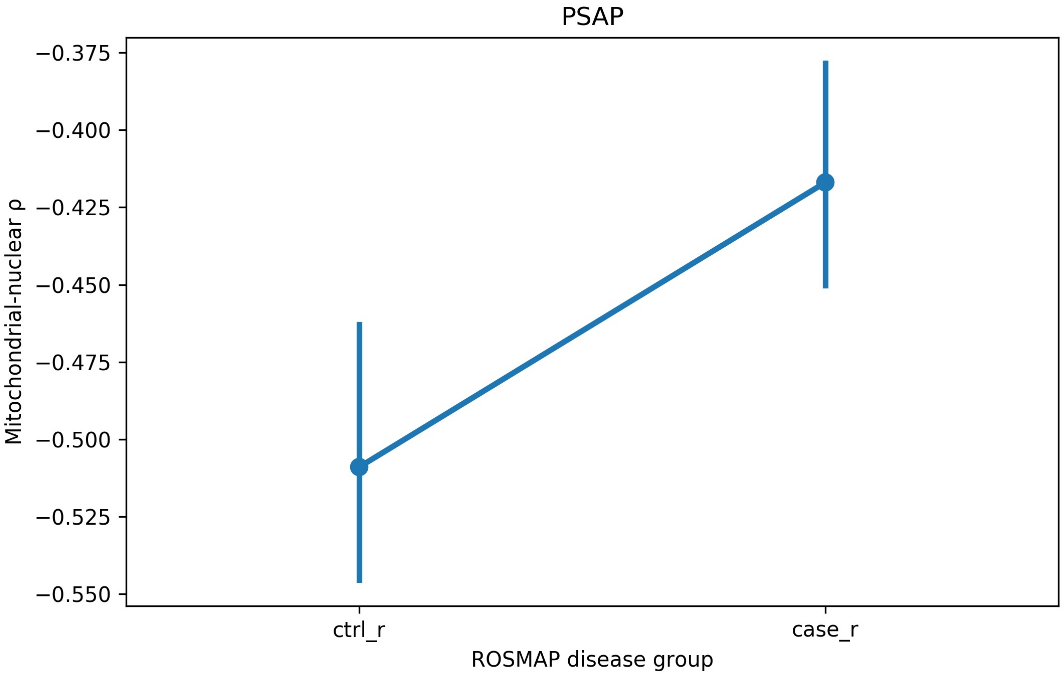

Supplementary figure 6. Heatmaps to show correlations (colourbar) and P-values (text) between covariates and principle components for **A.** The ROSMAP dataset and **B.** the GTEx frontal cortex data set

A

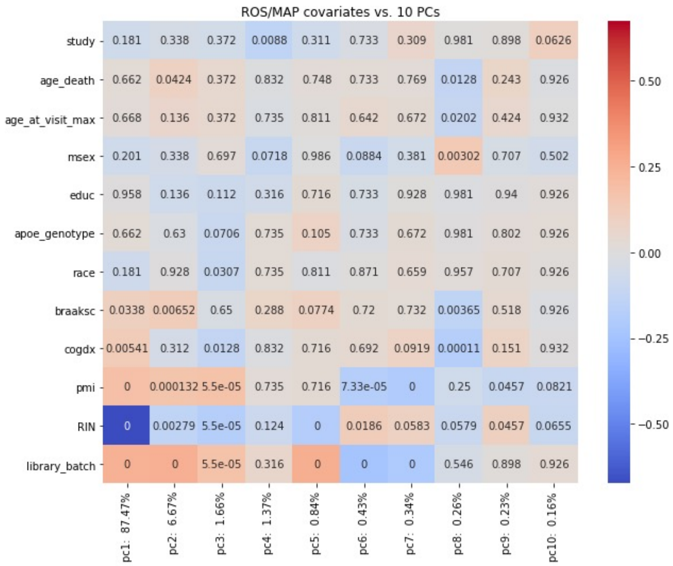

B

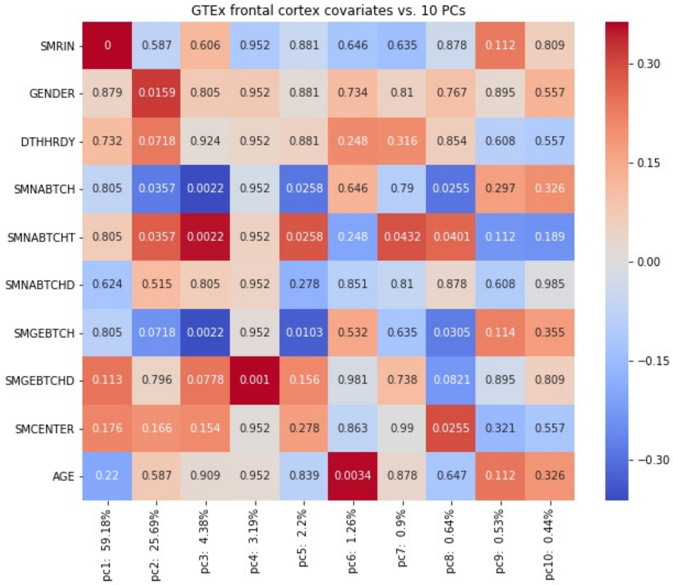

Supplementary figure 7. Side-by-side boxplots to visualise Scaden-derived cell type proportions of the ROSMAP data in control (blue) and case (orange) samples. Boxplots display the median, upper quartile (Q3) and lower quartile (Q1), with whiskers extending to Q3 + 1.5*IQR and Q1– 1.5*IQR. Diamond points represent outliers above Q3 + 1.5*IQR or below Q1– 1.5*IQR.

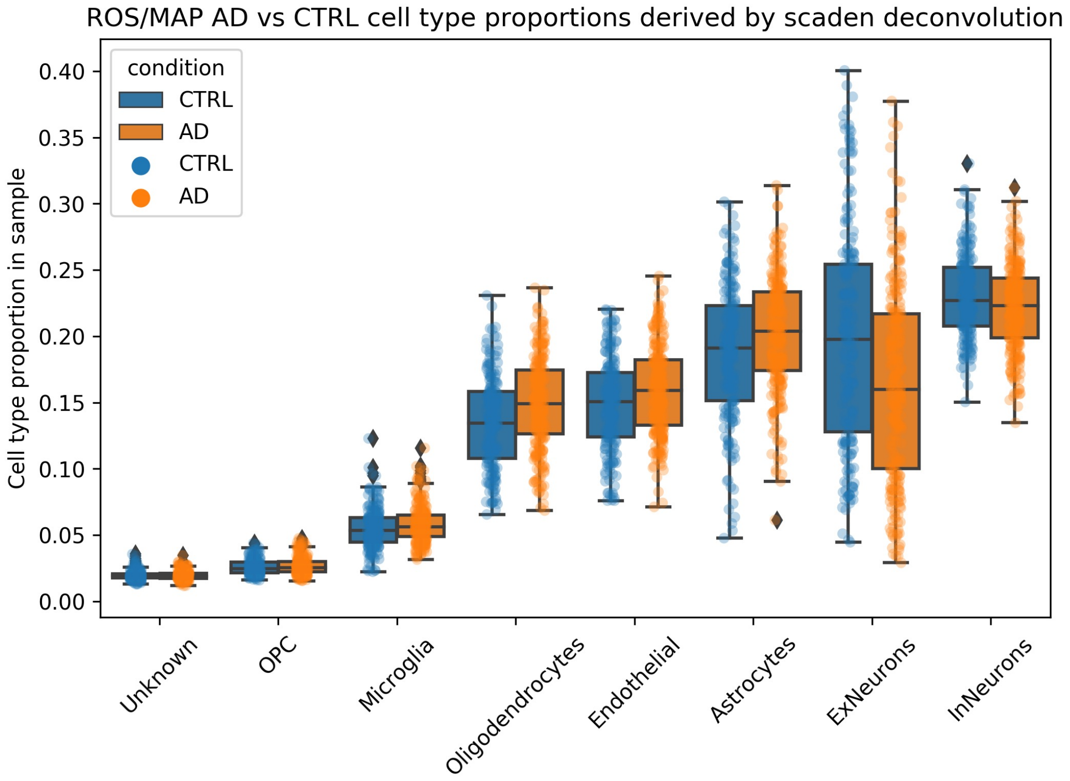

Supplementary Table 1. Full SynGO results for the high variance negative gene set

GO domain	GO term name	GSEA p-value
BP	process in the synapse	1.01E-27
CC	synapse	2.92E-22
CC	postsynapse	3.46E-20
CC	postsynaptic specialization	1.87E-18
CC	postsynaptic density	4.62E-18
CC	postsynaptic density membrane	1.49E-13
BP	trans-synaptic signaling	1.40E-11
BP	synapse organization	1.57E-11
BP	synaptic signaling	1.60E-11
BP	chemical synaptic transmission	8.45E-11
CC	presynapse	3.30E-08
CC	integral component of postsynaptic density membrane	6.30E-08
BP	modulation of chemical synaptic transmission	1.20E-07
CC	presynaptic membrane	3.19E-07
BP	process in the presynapse	2.55E-06
CC	extrinsic component of postsynaptic density membrane	2.96E-06
CC	integral component of presynaptic membrane	3.65E-06
BP	postsynaptic actin cytoskeleton organization	3.21E-05
BP	postsynaptic cytoskeleton organization	5.51E-05
CC	integral component of postsynaptic membrane	7.29E-05
CC	postsynaptic membrane	0.000148363
BP	regulation of postsynaptic membrane potential	0.000167015
BP	synaptic vesicle exocytosis	0.000837887
BP	postsynapse organization	0.000919204
CC	presynaptic active zone	0.001512649
CC	synaptic vesicle	0.002948953

Supplementary Table 2. Numbers of samples available for each GTEx tissue. N numbers of unique nuclear genes in the top 5% positive and negative of mitochondrial-nuclear gene correlations in each of the 12 GTEx CNS tissues

GTEx tissue	Tissue sample N number	Number of genes (+ and - Spearman's ρ) input into EWCE
Anterior cingulate cortex	80	470
Caudate basal ganglia	111	503
Cortex	104	340
Cerebellum	109	277
Cerebellar hemisphere	96	399
Frontal cortex	96	533
Hippocampus	88	426
Hypothalamus	85	268
Nucleus accumbens basal ganglia	100	487
Putamen basal ganglia	86	492
Spinal cord (cervical c1)	58	336
Substantia nigra	60	312

Supplementary Table 3. Numbers of samples available for each GTEx tissue, which also had available cell type proportion data as derived from Donovan et al..

GTEx tissue	Tissue sample N number
BrainAmygdala	53
BrainAnteriorcingulatecortexBA24	65
BrainCaudatebasalganglia	86
BrainCerebellarHemisphere	69
BrainCerebellum	89
BrainCortex	78
BrainFrontalCortexBA9	76
BrainHippocampus	67
BrainHypothalamus	62
BrainNucleusaccumbensbasalganglia	74
BrainPutamenbasalganglia	64
BrainSpinalcordcervicalc1	49
BrainSubstantianigra	43
